# The Cellular Origins of Cancer-Associated Fibroblasts and Their Opposing Contributions to Pancreatic Cancer Growth

**DOI:** 10.3389/fcell.2021.743907

**Published:** 2021-09-27

**Authors:** Paul Manoukian, Maarten Bijlsma, Hanneke van Laarhoven

**Affiliations:** ^1^Laboratory for Experimental Oncology and Radiobiology, Center for Experimental and Molecular Medicine, Cancer Center Amsterdam, Amsterdam UMC, University of Amsterdam, Amsterdam, Netherlands; ^2^Department of Medical Oncology, Cancer Center Amsterdam, Amsterdam UMC, University of Amsterdam, Amsterdam, Netherlands

**Keywords:** pancreatic ductal adenocarcinoma, stroma, heterogeneity, cancer-associated fibroblasts, cellular origins, resistance, radiation, pre-clinical models

## Abstract

Pancreatic tumors are known to harbor an abundant and highly desmoplastic stroma. Among the various cell types that reside within tumor stroma, cancer-associated fibroblasts (CAFs) have gained a lot of attention in the cancer field due to their contributions to carcinogenesis and tumor architecture. These cells are not a homogeneous population, but have been shown to have different origins, phenotypes, and contributions. In pancreatic tumors, CAFs generally emerge through the activation and/or recruitment of various cell types, most notably resident fibroblasts, pancreatic stellate cells (PSCs), and tumor-infiltrating mesenchymal stem cells (MSCs). In recent years, single cell transcriptomic studies allowed the identification of distinct CAF populations in pancreatic tumors. Nonetheless, the exact sources and functions of those different CAF phenotypes remain to be fully understood. Considering the importance of stromal cells in pancreatic cancer, many novel approaches have aimed at targeting the stroma but current stroma-targeting therapies have yielded subpar results, which may be attributed to heterogeneity in the fibroblast population. Thus, fully understanding the roles of different subsets of CAFs within the stroma, and the cellular dynamics at play that contribute to heterogeneity in CAF subsets may be essential for the design of novel therapies and improving clinical outcomes. Fortunately, recent advances in technologies such as microfluidics and bio-printing have made it possible to establish more advanced *ex vivo* models that will likely prove useful. In this review, we will present the different roles of stromal cells in pancreatic cancer, focusing on CAF origin as a source of heterogeneity, and the role this may play in therapy failure. We will discuss preclinical models that could be of benefit to the field and that may contribute to further clinical development.

## Introduction

The tumor microenvironment (TME) comprises both cellular and non-cellular components (Bremnes et al., 2011). It consists of a rich admixture of cells that harbor distinct activities and contribute differently to tumor growth and progression (Kiaris et al., 2004). In terms of cellular members, the stroma is mainly composed of fibroblast populations and other mesenchymal stromal cells, both of which are involved in forming connective tissue and extracellular matrix components; however, other cell types such as endothelial cells, pericytes, adipocytes, and immune cells also populate the TME (Valkenburg et al., 2018). Although most cells in the stroma possess certain tumor-suppressing capabilities, these cells are thought to be eventually coerced by the cancer cells and instructed to promote cancer growth, invasion, and metastasis (Valkenburg et al., 2018). Nevertheless, the exact contributions of stromal constituents involved in extracellular matrix (ECM) deposition and remodeling to cancer progression and therapy response have still to be fully understood. One unanimously agreed upon fact is that cancer-associated fibroblasts (CAFs) are prominent components of tumor stroma that have a large impact on nearly all aspects of cancer cell biology (Tao et al., 2017; Monteran and Erez, 2019). The roles of CAFs are quite extensive and will be discussed in following parts of the manuscript. These include the ability to: shape the ECM; modulate the innate and adaptive immune microenvironments; recruit and regulate leukocyte migration and inflammation via cytokines, chemokines, and growth factors; provide metabolic support (amino acids, lipids, and tricarboxylic acid cycle intermediates); and contribute to paracrine activation of mitogenic and pro-survival cellular signaling via cell surface receptor-ligand interaction and secreted proteins or exosomes (Krisnawan et al., 2020).

Initially, CAFs were assumed to be a homogeneous population of stromal cells. However, various studies have revealed heterogeneity within the CAF pool and identified that these cells can possess both pro- and anti-tumorigenic properties (Öhlund et al., 2014). Indeed, the diverse roles of CAFs have become evident in various cancer types giving rise to the notion of different CAF phenotypes based on morphological, behavioral, and functional properties (Allinen et al., 2004; Tchou et al., 2012; Liu et al., 2019b). This led to several studies with the aim of identifying different CAF subsets, their roles in the tumor microenvironment (TME), as well as their significance with regard to the clinic (Sahai et al., 2020). At first, two subsets of CAFs (iCAFs and myCAFs) were identified as the dominant fibroblastic populations in pancreatic cancer stroma (Öhlund et al., 2017). Shortly after that, an additional subpopulation, namely antigen-presenting CAFs (apCAFs), was identified and found to have antigen-presentation capabilities (Elyada et al., 2019). Importantly, there are some indications that certain CAF subsets derive from specific cell types (Helms et al., 2021), which may be relevant for the failure of recent targeting strategies. It is for this reason that investigating the subtype and cellular origin of CAFs has become relevant. Thus, in this review we will focus on CAFs, with emphasis on the concept of CAF origin as a source of intra-stromal heterogeneity and as the main culprit behind therapeutic shortcomings.

## The Cellular Origins of Cancer-Associated Fibroblasts

**FIGURE 1:**
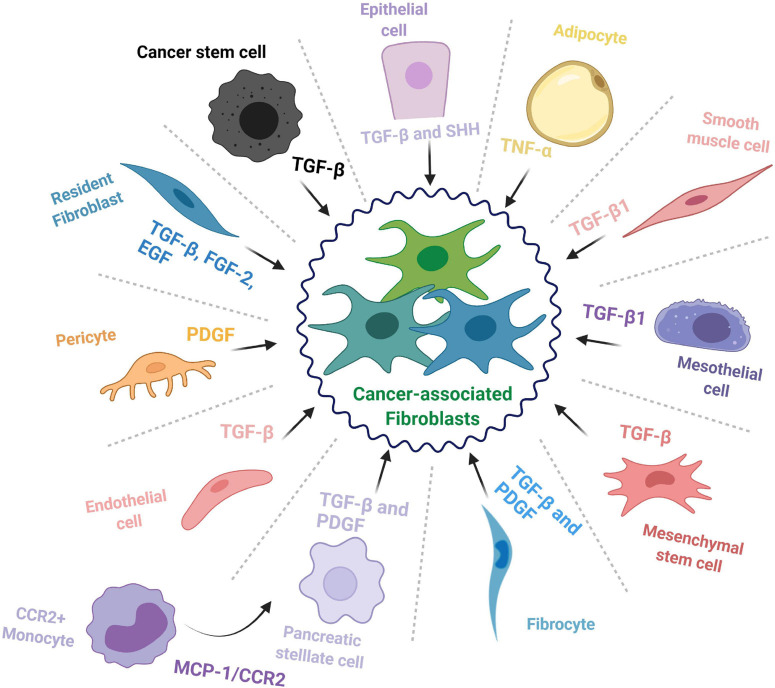
The cellular origins of cancer-associated fibroblasts. The cell types that contribute to the cancer-associated fibroblast (CAF) population and some of the major factors and signaling pathways involved in the transition toward a CAF phenotype.

In context of pancreatic cancer, CAFs commonly derive from three major sources: resident fibroblasts (Nair et al., 2017), pancreatic stellate cells (PSCs) (Vonlaufen et al., 2008), and tumor-infiltrating mesenchymal stem cells (MSCs) (Miyazaki et al., 2020); however, other cell types may also be recruited to enrich the CAF pool and feed the desmoplastic reaction (summarized in Figure 1).

### Resident Fibroblasts

Fibroblasts are mesenchymal cells with essential roles throughout embryonic development as well as adult organ function where they provide mechanical support and maintain tissue architecture (Di Carlo and Peduto, 2018). During normal physiological conditions, these cells typically remain in an inactivated state; however, they are involved in maintaining homeostasis and are activated to aid in wound repair for a short period, after which they revert to a quiescent-like state or are eliminated (McAnulty, 2007). Unfortunately, this is not always the case, as in a diseased state, these cells can be activated for an abnormally long period and lead to fibrosis, thereby impairing normal tissue function (Chan et al., 2019). A similar phenomenon occurs in cancer tissues, where fibroblasts (typically characterized by α-SMA expression) are perpetually activated and contribute to desmoplasia as cancer associated fibroblasts (McAnulty, 2007).

### Pancreatic Stellate Cells (PSCs)

Hepatic stellate cells were originally identified by Kupffer (1876) and mislabeled as a type of endothelial cell. They were later properly identified in 1952 and characterized two decades later as the major storage site of retinoids, and vitamin A homeostasis (Kawada, 1997; Pinzani and Gandhi, 2015). Subsequently, pancreatic stellate cells (PSCs) were identified in the mouse pancreatic duct in 1982 as a cell type that is enriched in lipid droplets and that has the capacity to store vitamin A (Watari et al., 1982). They were then observed in healthy sections from human and rat pancreas and named pancreatic stellate cells (Ikejiri, 1990). When activated from their resting state, PSCs adopt a myofibroblast-like phenotype and secrete various ECM components, thereby feeding into the CAF pool and promoting pancreatic fibrosis (Omary et al., 2007). However, beyond that, most of our knowledge on these cells is assumed from their resemblance to hepatic stellate cells (HSCs). Of note, stellate cells express mesenchymal, endodermal, as well as neuroectodermal markers (Kordes et al., 2012), which has complicated identifying their exact source. However, cell lineage tracing studies have confirmed that HSCs derive from mesenchymal cells and have evolved from a mesodermal origin (Cassiman et al., 2006; Asahina et al., 2011). It is worth mentioning that no such studies have been performed for their pancreatic counterparts, up till now, and little is known beyond the fact that the bone marrow can be a source of PSCs (Sparmann et al., 2010). Thus, the lineage of PSCs still needs to be mapped in full to reach a complete understanding of what these cells really are, how they influence the pancreatic TME, and how they contribute to different CAF populations in PDAC.

### Mesenchymal Stem Cells (MSCs)

Mesenchymal stem cells are multipotent adult progenitor cells that were initially discovered in the bone marrow but were later documented in multiple other tissues, including umbilical cord and fat tissue (Pittenger et al., 1999). Mesenchymal stem cells hold self-renewal properties and the ability to differentiate into multiple tissues/cell types including bone, cartilage, muscle and fat cells, as well as connective tissue (Ding et al., 2011). Recent studies have introduced the possible role of MSCs during inflammation, immune response, wound healing, and cancer progression (Chamberlain et al., 2007). Current knowledge suggests that MSCs are recruited into pancreatic tumors where they contribute to disease progression and facilitate cancer therapy resistance (Saito et al., 2018). As a matter of fact, MSCs have been proposed as potential delivery vehicles for anticancer agents in the clinic for many cancer types, including PDAC, due to their ability to home toward tumor sites (Spano et al., 2019). Naturally, it is for this specific property that MSCs are considered one of the main sources of CAFs; for instance, bone marrow-derived MSCs are recruited to PDAC tumors where they differentiate into CAFs or tumor-associated MSCs (TA-MSCs), which can act as yet another source of CAFs and further enrich the population (Liu et al., 2019a). However, it is not yet known how differently sourced MSCs contribute to CAF subsets in PDAC. A recent study showed that adipose tissue-derived MSCs could differentiate into two different CAF subpopulations: direct contact co-culture with a PDAC cell line could induce their differentiation toward either myCAF or iCAF phenotype, while an indirect co-culture induced differentiation into only iCAFs (Miyazaki et al., 2021). This sheds light on the role of MSCs in feeding the CAF population, but whether this is the case for MSCs from other sources as well needs to be studied.

### Cells From Non-fibroblastic Lineage

Besides the abovementioned sources, CAFs have been documented to transdifferentiate from seemingly unrelated cell types such as epithelial cells (Iwano et al., 2002; Kalluri and Weinberg, 2009), endothelial cells (Zeisberg et al., 2007), adipocytes (Bochet et al., 2013), pericytes (Hosaka et al., 2016), mesothelial cells (Rynne-Vidal et al., 2015; Koopmans and Rinkevich, 2018), and smooth muscle cells (McAnulty, 2007). Further, fibrocytes, a circulating mesenchymal cell population of monocytic origin, may contribute to the pool of CAFs in the TME (Reilkoff et al., 2011; Gunaydin et al., 2015). A recent study reported that CCR2+ monocytes can migrate to the pancreas following activation through MCP-1/CCR2 signaling and differentiate into PSCs (Ino et al., 2014). Another interesting source of CAFs is the cancer stem cell (CSC) population; indeed, in some cancer types, CSCs have been documented to adopt a myofibroblast-like phenotype, by undergoing EMT, and subsequently contribute to tumor growth (Petersen et al., 2003; Huang et al., 2015). This shows how deep the repertoire of CAF progenitors may be and underscores the need for comprehensive studies on the relation between CAF origin and function.

## The Contributions of Single Cell Sequencing (sCRNA-Seq) Technology to Cancer-Associated Fibroblast Identification

In a recent time-course scRNA-seq study, fibroblasts from pre-invasive human and mouse pancreatic lesions were analyzed to shed some light on gradual changes during pancreatic tumorigenesis and possibly the origin of such cells (Schlesinger et al., 2020). A mouse model with inducible expression of Kras-G12D was used to profile the changes in stromal and acinar cells during the progression from preinvasive lesions to PDAC. Two clusters of fibroblasts were initially observed (Igfbp5+ cells and Il6+ cells). Interestingly, however, during the late stages, Il6+ cells expressed high levels of cytokines (including Ccl2, Ccl7, and Cxcl2) and were reminiscent of inflammatory CAFs (iCAFs). Whereas myofibroblasts (Acta2-positive cells) expressing high levels of Des and Igfbp5 compared to the IL6+ faction were observed. Further, three additional subpopulations of fibroblasts expressing distinct sets of genes were apparent: (i) proliferating fibroblasts (most cells expressed Acta2); (ii) fibroblasts that were recently discovered as MHC-II positive and expressed additional related genes such as CD74 and CD8323; as well as (iii) 15 months’ CAFs, which clustered uniquely, that expressed Acta2, Tgfb1, and Cx3cl1, and members of the Wnt signaling pathway, such as Wnt265.

Another single cell transcriptomic study also investigated the biology of primary PDAC tumors as well as metastatic lesions from human patients (Lin et al., 2020). A noteworthy observation was that cells from primary PDAC tumors clustered into seven major populations whereas those from metastatic lesions clustered into three, which directly revealed that the two settings are more dissimilar than alike and calls for more focused studies. Interestingly, unsupervised clustering of CAFs from the primary tumors led to three major clusters (dubbed as c0, c1, and c2), which, in contrast to tumor cells, did not cluster by patient. This finding indicated that CAFs from different patients were more similar in their gene expression profiles than their matching tumor cell populations. The team also set out to determine whether the CAF clusters identified in their analysis matched the three classical CAF subtypes. Only one cluster (c0) was enriched for previously described (myCAF) markers, whereas the remaining two clusters were not enriched in signature genes associated with either iCAFs or apCAFs. In fact, it was found that the signature genes that define cluster 1 were more enriched with those associated with quiescent CAFs; while cluster 2 displayed an expression signature that resembles that of smooth muscle cells, which drove the authors to postulate that these cells might be mural cells including pericytes and vascular smooth muscle cells from the blood vessels.

A recent scRNA-seq paper by Chen K. et al. (2021) described a novel subgroup of CAFs with complement-secreting capacity (csCAFs) in human PDAC tumors that, despite their resemblance to iCAFs, qualified as a unique subgroup. Subsequently, the group demonstrated the existence of these cells and their location by performing by RNA ISH (RNA *in situ* hybridization) and IF (immunofluorescence) on human PDAC sections with different clinical stages. The findings of this study suggested that csCAFs may play a tumor-suppressive role in PDAC microenvironment and that this population is gradually lost during tumor progression.

Another prominent study described a mass cytometry approach that allowed the analysis of mesenchymal stroma in both normal and tumor murine pancreatic tissues (Hutton et al., 2021). The findings of this study revealed extensive stromal heterogeneity across both tissues and led to the identification of coordinated relationships between mesenchymal and immune cell subsets in PDAC. Remarkably, it was found that the expression of CD105 could distinguish two stable and functionally distinct pancreatic fibroblast lineages in both human and murine settings. It was also evident that the CD105-positive fibroblast population is tumor-permissive, while CD105-negative fibroblasts are highly tumor suppressive in a manner entirely dependent on functional adaptive immunity.

Recent advances in single cell technology, including scRNA-Seq as described above, have partly overcome previous shortcomings in the identification of CAF subsets. Nonetheless, the information available in literature regarding CAF *origin* is still somewhat limited. This underscores the need for more comprehensive approaches that make good use of scRNA-Seq findings, perhaps together with lineage tracing technologies to better understand how tumor stroma is shaped and identify the contributions and roles of different CAF progenitor cells in PDAC tumors. A recent paper highlights the importance of such tracing studies for understanding PDAC; targeted ablation specifically of PSC-derived CAFs revealed non-redundant functions for this defined CAF population in shaping the PDAC microenvironment (Helms et al., 2021). This finding links stromal evolution from distinct cells of origin to transcriptional heterogeneity among PDAC CAFs and demonstrates unique functions for CAFs of a defined cellular origin, further supporting the notion of cellular origin being a driver of CAF heterogeneity and urging more studies.

## Tumor Cell-Derived Signals Involved in Fibroblast-Like Cell Recruitment and Activation

Numerous studies have focused on the aspect of tumor-stroma interactions in PDAC. Crosstalk between pancreatic cancer cells and the surrounding stroma is complex, but many key elements in this process have now been identified (Valkenburg et al., 2018). The main mechanisms of stroma cell-tumor cell interaction include the exchange of extracellular molecules, such as extracellular vesicles, cytokines, and chemokines. Other means of communication, such as direct cell-cell interaction, have also been shown to influence the recruitment and activation of CAFs (Sperb et al., 2020). Finally, the roles of key intracellular signaling pathways such as JAK/STAT, mTOR, Sonic Hedgehog (SHH), and NF-kB are relatively well defined in the context of PDAC-stroma crosstalk (Rhim et al., 2014; Duluc et al., 2015; Wörmann et al., 2016; Garg et al., 2018).

**FIGURE 2:**
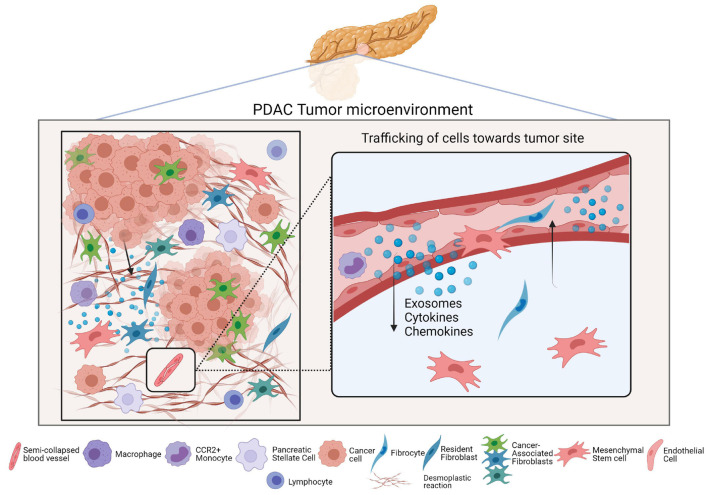
Cell trafficking and the PDAC tumor microenvironment. The **left** panel of the figure is a simplified depiction of the PDAC tumor microenvironment and includes the major cell types that reside in it. The **right** panel is a magnified view of how different secreted factors serve to recruit CAF progenitors into PDAC tumors wherein they enrich the CAF population.

It is well established that cancer cells secrete factors like chemokines to recruit inflammatory cells, MSCs, and PSCs toward tumor sites, as well as to instruct them to create a nurturing environment that encourages tumor progression (Figure 2; Roy et al., 2017; Geismann et al., 2019). One example study demonstrated that in a coculture setting with Panc-1 cells, normal skin fibroblasts were driven to secrete collagens (I and III), PDGF, as well as fibronectin, thereby contributing to a more desmoplastic environment (Mahadevan and Von Hoff, 2007). The authors went further to identify that transforming growth factor beta 1 (TGF-β1) and fibroblast growth factor (FGF)-2 were involved in this phenomenon and led to the proliferation of both cell types. Typically, many of the signaling processes in the stroma influence the behavior of the surrounding cancer cells as well. In fact, increased TGFβ signaling in the TME seems to form an autocrine-paracrine loop that serves to promote invasion and metastasis of tumor cells during later stages of many cancers, including PDAC (Bierie and Moses, 2006).

Various processes are involved in the transition of stromal cells toward a CAF phenotype and are typically dependent on stimuli such as local hypoxia, oxidative stress, and growth factor release. Indeed, certain factors including TGF-β, epidermal growth factor (EGF), platelet-derived growth factor (PDGF), and fibroblast growth factor 2 (FGF2) are considered key regulators of fibroblast recruitment and activation (Karagiannis et al., 2012; Wu et al., 2021). In pancreatic tumors, PSCs can become myofibroblast-like and express α-SMA upon activation by growth factors (TGF-β and PDGF), inflammatory cytokines (TNF-α, IL-1, IL-6, IL-8, IL-10, etc.), as well as other factors such as EMMPRIM, ET-1, Angiotensin II, SHH, and further enrich the CAF pool (Heinemann et al., 2014; McCarroll et al., 2014). Furthermore, once activated, PSCs perpetuate their own activity via autocrine loops, which in turn promotes vicious stromal development and ECM deposition (Erkan et al., 2012).

The SHH pathway is an important signaling axis in PDAC as it is involved in pancreatic fibrogenesis (Jung et al., 2011). Initially, multiple lines of evidence indicated that blockade of this pathway with small-molecule inhibitors can inhibit the development of pancreatic tumors (Bai et al., 2016). This attracted a great deal of attention in the pancreatic cancer field and proposed novel therapeutic regimens. Nonetheless, these inhibitors have yielded rather contradictory findings. Several clinical trials (NCT01064622; NCT01088815) have investigated the efficacy of SHH inhibition in pancreatic cancer but overall no significant benefits were observed, despite promising pre-clinical findings (Olive et al., 2009; Özdemir et al., 2014). Since then, papers have emerged that argue against Hedgehog (Hh) pathway inhibition as a therapeutic approach in PDAC. Preclinical studies have shown that genetic and pharmacological inhibition of Hh pathway activity in fact accelerates PDAC progression (Lee et al., 2014). One study described the role of Shh signaling in driving the formation of a fibroblast-rich desmoplastic stroma in PDAC (Rhim et al., 2014). Indeed, the findings of this study show that not only did Shh-deficient tumors have reduced stromal content, but that they were, surprisingly, more aggressive and exhibited undifferentiated histology, increased vascularity, and heightened proliferation. This was an indication that Hedgehog-driven stroma may have a suppressive role in PDAC. This was then linked to the findings of other studies wherein depletion of CAFs and subsequent fibrosis could induce immunosuppression and accelerate pancreatic cancer progression (Özdemir et al., 2014). A recent paper shed further light as to why Shh inhibition may not be the most ideal approach for PDAC treatment (Steele et al., 2021). The findings of this study showed that Shh signaling was specifically activated in myCAFs and that inhibiting this pathway using the smoothened antagonist LDE225 inhibited Shh signaling, reduced myCAF numbers, and increased iCAF numbers in a PDAC mouse model, resulting in an immune suppressive microenvironment.

Epigenetic regulation of normal fibroblasts has been recently highlighted as a means of conversion into CAFs in different cancers (head and neck, lung, and breast). Mechanistically, exposure of the fibroblasts to pro-inflammatory leukemia inhibitory factor (LIF) triggers an epigenetic switch leading to constitutive activation of Janus kinase 1–signal transducer and activator of transcription 3 (JAK1–STAT3) signaling, which is sustained by the DNA methyltransferase DNMT1, and activates the fibroblasts into pro-invasive CAFs with increased acto-myosin contractility (Albrengues et al., 2015); this suggests a shift toward a myCAF-like phenotype but goes against previous findings that linked the iCAF and myCAF phenotypes with JAK/STAT and TGF-β signaling, respectively (Biffi et al., 2019; Chen and Song, 2019). Lactate-mediated epigenetic reprogramming has also been noted in human pancreatic cancers where it regulates the formation of CAFs (Bhagat et al., 2019); in fact, epigenetic reprogramming, in the form of widespread loss of DNA methylation and gain of cytosine hydroxymethylation at selective promoters, was observed in both MSC-derived and primary (patient-derived) pancreatic CAFs and linked the process to increased alpha-ketoglutarate production.

It is worth noting that pancreatic cancer cells can use extracellular vesicles (see also paragraphs on *exosomes* below) to educate members of the TME and contribute to carcinogenesis (Stefanius et al., 2019). One study assessed the ability of pancreatic cancer to recruit PSCs *in vitro* by performing Transwell assays and *in vivo* using a mouse model (Zhang et al., 2019). The group showed that pancreatic cancer cell-derived exosomes containing Lin28B (Exo-Pan and Exo-Mia) promoted the recruitment of PSCs by activating the Lin28B/let-7/HMGA2/PDGFB signaling pathway, which highlights the importance of exosomal signaling in cancer.

Importantly, specific cancer cell genotypes can differently instruct CAFs. One team analyzed tumors harvested from two mouse models with different metastatic propensities and saw that p53 mutant PDAC cells can drive CAFs into establishing a prometastatic and chemoresistant microenvironment due to increased nuclear factor-κB (NF-κB) signaling and high levels of the NF-κB target gene tumor necrosis factor-α (TNF-α) (Vennin et al., 2019). Another finding from this study was that p53 mutant-educated CAFs could induce the invasion of p53 null cancer cells, which are normally considered poorly invasive, supporting the notion that CAFs may play a role in mobilizing aggressive cells within the tumor (Sperb et al., 2020). Similarly, another group identified a unique, highly rigid, matricellular-stromal (also described as mesenchymal-like) phenotype in PDAC (linked to integrins, YAP, and SOX2) that results in reduced epithelial TGF-β signaling and elevated tumor cell contractility as well as tumor progression and aggression (Laklai et al., 2016).

In conclusion, we highlighted how pancreatic cancer cells shape the TME by recruiting and educating CAFs from different sources/precursor cells and presented the main signaling pathways that are involved in these phenomena and how they dictate CAF subtype. We also went further and stressed the importance of cancer cell genotype in further increasing heterogeneity in the CAF pool and generating CAFs that encourage PDAC cell aggressiveness and support tumor progression.

## Cancer-Associated Fibroblast-Derived Signals That Support Pancreatic Cancer

Cancer-associated fibroblasts are a substantial source of signals that can promote tumor growth and impact on therapy responses (Chen et al., 2014; Ali et al., 2015; Richards et al., 2017). CAFs can foster tumor cell growth, angiogenesis, and invasion by secreting paracrine factors, such as pro-inflammatory cytokines, chemokines, prostaglandins (PGE), growth factors, as well as proteases, and by remodeling the extracellular matrix (ECM) (Chan et al., 2019). Moreover, the production of TGFβ, leukemia inhibitory factor (LIF), growth arrest-specific protein 6 (GAS6), fibroblast growth factor 5 (FGF5), growth differentiation factor 15 (GDF15), and hepatocyte growth factor (HGF) promotes invasive and proliferative behavior in cancer cells (Sahai et al., 2020). VEGF-A and Tenascin-C are other relevant markers in context of stromal contribution to cancer progression; the expression of the former is a driver of angiogenesis and metastatic colonization, while that of the latter provides cancer cells protection from apoptosis (O’Connell et al., 2011). PSCs have also been documented to influence cancer cells through hypoxia-inducible factor 1α (HIF-1 α)-mediated signaling and thereby leading to the subsequent activation of various genes involved in cell survival, progression, invasion, and metastasis (Omary et al., 2007). One study identified a subpopulation of CAFs designated as cancer-associated mesenchymal stem cells (CA-MSCs) and demonstrated that they contribute to cancer invasion via granulocyte-macrophage colony-stimulating factor (GM-CSF) secretion (Waghray et al., 2016). These findings were confirmed by another study which identified that the mechanism underlying GM-CSF-induced invasion is related to downregulation of E-cadherin and upregulation of TWIST1 (which are associated with resistant cancer cell phenotypes) and vimentin via the JAK2/STAT3 pathway (von Ahrens et al., 2017).

Another method by which fibroblasts interact with tumor cells is the exchange of metabolites. Many recent studies have focused on metabolites and their importance in tumor-stroma interaction. Here, we will briefly mention some examples of how stromal cells support pancreatic cancer growth by providing metabolites. For instance, fibroblasts are known for undergoing autophagy in response to stimulation by cancer cells and thereby releasing alanine, which subsequently contributes to pancreatic cancer cell energy production by feeding the tricarboxylic acid (TCA) cycle (Sahai et al., 2020). PSC-derived alanine can also promote PDAC cell growth in nutrient-limited conditions by acting as an alternative carbon source to glucose and glutamine and fueling the TCA cycle, supporting lipid and non-essential amino acid biosynthesis, as well as shunting glucose for serine/glycine biosynthesis (Xu et al., 2020).

Many recent studies have been aimed at studying the role of extracellular vesicles, such as exosomes, in tumor-stroma interaction (Doyle and Wang, 2019). Exosomes are small membrane-enclosed vesicles (<150 nm) of endosomal origin that contain numerous molecular components including proteins, mRNAs, and miRNAs (Ruivo et al., 2017). Exosome secretion has been proven to play a crucial role in long-distance communication in various tissue types (Dai et al., 2020). MSCs and fibroblasts seem to be a rich source of exosomes and have been recorded to interact with tumor cells and participate in tumorigenesis as well as tumor progression (Hu et al., 2015; Yin et al., 2019). For instance, miR-142-3p transfer via BM-MSC-derived exosomes, as well as those from fibroblasts, has been noted to increase the CSC population in colorectal cancer (Li and Li, 2018). CAFs also seem to protect pancreatic cancer cells from gemcitabine treatment via exosomal Snail and miR-146a that enhance the proliferative capacity of the cancer cells and induces EMT, which is linked with resistance and metastasis (Richards et al., 2017); interestingly, exosome secretion was upregulated as a result of gemcitabine treatment, which highlights the importance of CAFs in *acquired* pancreatic cancer resistance. Many efforts aimed to explore the secretome of CAFs and various secreted factors and active substances have been identified to be involved in tumor-stroma crosstalk and might serve useful to better understand CAF heterogeneity and establish effective treatment approaches.

## The Tumor-Suppressive Potential of Cancer-Associated Fibroblasts in PDAC

The notion of a tumor-suppressive population of CAFs has gained a lot of attention. This led to the proposal of markers, such as α-smooth muscle actin (SMA), as candidates to identify tumor-restraining CAFs in PDAC. However, other studies had already revealed a correlation between the number of α-SMA+ CAFs and poor outcome in various types of human solid cancers, leading to an opposing hypothesis which states that α-SMA+ CAFs are in fact tumor-permissive and tumor-promoting (Miyai et al., 2020). Interestingly, a recent study provided insight into the nature of tumor-restraining CAFs and suggested that they may share molecular properties with PSCs and MSCs (Mizutani et al., 2019). Indeed, markers that are representative of PSCs and MSCs seem to be associated with favorable prognosis in PDAC; for instance, the number of CD271+ stellate cells is associated with good prognosis in pancreatic cancer (Fujiwara et al., 2012). CD36-expressing fibroblasts with a low expression of CD36 contribute to the deposition of collagens and fibronectin, to a higher degree than their highly CD36-expressing counterparts do, thereby contributing to a more desmoplastic environment. The expression of Meflin, a glycosylphosphatidylinositol-anchored protein as a marker of mesenchymal stromal/stem cells, in CAFs has also been correlated with favorable outcome in human PDAC (Maeda et al., 2016). Remarkably, Meflin has the capacity to suppress αSMA expression (myofibroblastic differentiation) in CAFs as well as ECM remodeling, a crucial process for cancer progression, which might give some insight into the functionality of tumor-suppressive CAFs and how they inhibit tumor growth. Of course, more comprehensive studies in genetic animal models are necessary to confirm these *in vitro* findings and whether targeted approaches could be safe and efficacious in the clinic.

There, thus, appears to be a link between CAFs expressing markers that are typically attributed to MSCs and/or PSCs and them being tumor-repressive. However, since MSCs and PSCs are major sources for CAFs, this suggests one of two scenarios; (i) that tumor-repressive CAFs are less activated and therefore more similar to MSCs/PSCs, or (ii) that there is heterogeneity among MSCs/PSCs that persists after they become CAFs. These notions seem less farfetched when you take into account the findings of Waterman et al. (2012) who showed that MSCs can have two phenotypes that present opposing effects on cancer growth and metastasis.

Exosomes are also involved in mediating the anti-cancer effects of stromal cells in pancreatic cancer. One study demonstrated that BMSC-derived exosomes could suppress proliferation, invasion, and metastasis as well as promote apoptosis in pancreatic cancer cells by transferring miR-126-3p and, thereby, downregulating ADAM9 (Wu et al., 2019). Another study showed that low miR-1231 expression in peripheral blood-derived exosomes was significantly correlated with the TNM stage of pancreatic cancer, hinting toward a potentially inhibitory effect of exosomal miR-1231 on the occurrence and development of the disease (Shang et al., 2019).

To sum up, we know that certain aspects dictate whether CAFs exert tumor-suppressive or -promoting activities and that this needs to be fully understood in order to improve therapeutic regimens and/or adopt more targeted approaches. Taking the abovementioned information into account, there seems to be a link between the tumor-suppressive activity of CAFs and them expressing PSC/MSC markers and that tumor-suppressive CAFs seems to have reduced α-SMA expression and ECM remodeling abilities compared to their tumor-promoting counterparts. We propose that more advanced *ex vivo* or animal models should be utilized in order to corroborate these findings and make way to clinically relevant targeted approaches that target specific CAF subpopulations in PDAC. We will discuss this in a subsequent section that discusses available technologies and how they may be utilized for improving PDAC modeling.

## The Role of the Stroma in Conferring Resistance to Chemotherapy

Cancer-associated fibroblasts, ECM components, as well as immune cells can all directly confer a resistant phenotype in tumor cells (Krisnawan et al., 2020), here, we will mainly discuss the role of CAFs in this regard. Many studies have identified these cells as promoters of resistance; however, the molecular mechanisms underpinning these phenomena remain unclear. One group demonstrated that CAF-secreted SDF-1 drives gemcitabine resistance in pancreatic cancer by forming a positive feedback loop that drives paracrine induction of SATB-1 in the pancreatic cancer cells (Wei et al., 2018). Another team identified the role of miR-21 expression in CAF activation, through PDCD4 upregulation, as well as resistance to gemcitabine using tumor samples from PDAC patients (Zhang L. et al., 2018). This led to some mechanistic insights as the authors revealed that high miR-21-expressing CAFs secreted elevated levels of MMP-3, MMP-9, PDGF, as well as CCL-7, thereby promoting the invasion of PDAC cell lines and mediating gemcitabine resistance in an *in vivo* setting. USP27X is another interesting candidate in context of stroma-mediated chemoresistance. In fact, USP27X is activated by TGFβ, a known inducer of EMT (Shen et al., 2017), and plays a major role in both TGFβ-induced EMT and fibroblast activation (Lambies et al., 2019), which suggests it contributes to a positive activation loop. The IL-1β-IRAK4 signaling pathway is yet another major player in PDAC cell response to chemotherapy (Zhang D. et al., 2018); CAFs robustly express IRAK4 and NF-κB and support PDAC cell chemoresistance. Interestingly, this axis may be a valuable therapeutic target to potentially circumvent chemoresistance in pancreatic cancer as targeting IRAK4 or IL-1β could render PDAC tumors less fibrotic and more sensitive to gemcitabine and, potentially, other chemotherapeutic agents (Zhang D. et al., 2018; Elahi-Gedwillo et al., 2019).

Another group showed that Periostin, which is exclusively expressed by PSCs, confers resistance to gemcitabine in pancreatic cancer cells (Dauer et al., 2017). PSCs have also been reported to promote the expression of HES1 in pancreatic cancer cells, thereby making them more resistant to chemotherapy. The SDF1/CXCR4 pathway is a major signaling axis that contributes to resistance in pancreatic cancer; SDF1 is secreted by CAFs and interacts with CXCR4, its receptor, which is present on the cancer cells, and induces gemcitabine chemoresistance in these cells by paracrine-induced activation of the intracellular FAK-AKT and ERK1/2 signaling pathways and a subsequent autocrine IL-6 signaling loop (Zhang et al., 2015). Interestingly, IL-6 was also found to induce the production of Survivin, an inhibitor of apoptosis, in cancer cells (Duluc et al., 2015); the authors also identified that mTOR/4E-BP1 signaling is activated in cancer cells as a response to CAF-secreted factors and plays a role in imparting chemoresistance. Another interesting finding was that CAFs serve as a source of CYR61 in co-culture models and also induce chemoresistance by downregulating the nucleoside transporters hENT1 and hCNT3, which are known to mediate cellular uptake of chemotherapeutic drugs such as gemcitabine (Hesler et al., 2016).

Pancreatic stellate cells also contribute to shaping a hypovascular and hypoxic microenvironment, which is a characteristic of pancreatic tumors (Margetis and Drekolias, 2015) and a major obstacle for the delivery of chemotherapeutics (Sheth et al., 2013). These features intensify chemoresistance and encourage fibrosis in a self-perpetuating hypoxia-fibrosis cycle (Margetis and Drekolias, 2015). This not only promotes EMT and genetic instability in cancer cells, but also impairs drug delivery (McCarroll et al., 2014); it is worth noting that stromal depletion prior to chemotherapy administration could enhance intratumoral drug perfusion, rendering tumors vulnerable to cytotoxicity and, thereby, inhibiting tumor growth and prolonging overall survival. However, removing the stroma, which acts as a tumor-containing fibrotic barrier, may have also unintentionally encouraged the metastatic evolution of aggressive clones (Özdemir et al., 2014), which might explain the disappointing results of a phase II clinical trial following the paper initially describing this concept (Olive et al., 2009). Interestingly, PSCs survive in patients treated with full-dose gemcitabine plus concurrent hypo-fractionated stereo-tactic radiosurgery, and display a more activated phenotype following this regimen (Cabrera et al., 2014). In addition to contributing to mechanical properties and hypoxia-induced chemoresistance, PSCs can directly impact cancer cell response to chemotherapy; PSC secretions have been shown to confer a chemoresistant phenotype in pancreatic cancer cells by suppressing H2O2-induced apoptosis (Vonlaufen et al., 2008) in addition to decreasing pancreatic cancer cell sensitivity to gemcitabine, 5-fluorouracil (5-FU), cisplatin, doxorubicin as well as radiation therapy (Miyamoto et al., 2004; Hwang et al., 2008).

Despite the current knowledge, however, there are not enough comprehensive studies on the roles of different CAF subsets with respect to pancreatic cancer cell sensitivity to chemotherapy, and whether certain subsets exist that strictly alleviate resistance. In fact, most of the available data on resistance cannot be correlated with CAF subtype due to the lack of expression data for relevant markers such as α-SMA or IL-6. We should also mention that most studies that are currently published tackle gemcitabine resistance but there are not enough studies on more recently implemented regimens such as 5-FU, leucovorin, irinotecan and oxaliplatin (FOLFIRINOX) (Chiorean and Coveler, 2015). This is something to acknowledge and warrants further research.

## The Tumor Stroma and Radioresistance

Radiation therapy has a prominent place in treating locally advanced pancreatic cancer (Hall and Goodman, 2019). Unfortunately, however, the molecular pathways that contribute to resistance to ionizing radiation (IR) in pancreatic cancer remain poorly understood (Brunner et al., 2005; Kimple et al., 2010). Much evidence has emerged to support the role of PSCs and CAFs as major contributors to radio-resistance (Krisnawan et al., 2020). These cells were shown to confer resistance by multiple modes of action. On the one hand, they could achieve this feat by direct contact with surrounding cancer cells mainly via β-Integrin-FAK signaling (Mantoni et al., 2011). On the other hand, they could contribute to a resistant cancer phenotype by secreting a plethora of factors (Linares et al., 2021).

A recent study described the role of stromal fibrosis in activating pro-survival and epithelial-to-mesenchymal transition (EMT) pathways in PDAC. The group identified two cell-surface proteins, a disintegrin and metalloprotease 10 (ADAM10) and ephrinB2, as drivers of fibrosis and tumor progression after radiation therapy (RT) and suggested that activation by ephrinB2 drives fibroblasts toward a myofibroblast differentiation, thereby driving cancer invasion (Mueller et al., 2021). Unfortunately, there are few studies that include data on fibroblast heterogeneity and/or phenotype with respect to resistance to RT and most studies focus more on the mechanisms underpinning RT resistance in general; as such, this section will be a more general viewpoint on the role of CAFs in mediating/alleviating radioresistance in PDAC. We will also use this opportunity to encourage more studies on different CAF populations and subsets and how they are affected by RT and by which underlying mechanisms.

As was introduced above, exosome transfer is a prominent mechanism for tumor-stroma signaling. This mode of communication is also implicated in conferring resistance to radiation (Zhang H. et al., 2018). For instance, paracrine anti-viral RIG-I and juxtacrine NOTCH (NOTCH3-JAG1) have both been identified as contributors to therapy-resistance, which they facilitate by inducing tumor-initiating cell expansion in a STAT1-dependent fashion (Boelens et al., 2014). Exosomal lipids have been shown to induce drug resistance in MiaPaCa-2 cells, via the C-X-C motif chemokine receptor 4 (CXCR4)/stromal cell derived factor (SDF)-1α signaling axis (Yan et al., 2018). Some studies have indicated that exosomes can increase intracellular ROS levels in pancreatic cancer cells, thereby making them more susceptible to DNA-damage and radiation-induced killing. Mechanistically, these effects were linked to miR-6823-5p, within exosomes originating from irradiated cells, which contributed to modulating superoxide dismutase 1 (SOD1) levels (Nakaoka et al., 2021).

Another study highlighted the important role of tumor stroma in hampering or even negating the beneficial effects of radiotherapy in PDAC treatment (Zhao et al., 2015). Specifically, the authors demonstrated the effectiveness of Cyclopamine, a SHH pathway inhibitor, and its promise as a radio-sensitizing and stromal disruptive agent in PDAC treatment. Interestingly, this seems contradictory to other studies wherein targeting SHH in PDAC was found to contribute to an immune-suppressive environment (Steele et al., 2021).

Cancer-associated fibroblasts and bone marrow cells have been noted to protect breast cancer cells by inducing interferon (IFN)-related DNA damage-resistance in a STAT1-dependent manner, leading to radio-resistance (Boelens et al., 2014). Although the effect of IFN-γ on resistance has not yet been explored in pancreatic cancer, its role in inhibiting the growth of, as well as tumor-associated macrophage trafficking in, pancreatic cancer has already been established (Detjen et al., 2001; Zhang et al., 2020); thus, the aforementioned concept is worth exploring in PDAC and might be a successful strategy to improve the efficacy of radiation-based combinatorial regimens.

Similarly, conditioned medium from PSCs led to a dose-dependent induction of pancreatic cancer cell proliferation, migration, invasion, and colony formation and caused resistance to gemcitabine and RT (Hwang et al., 2008). The mechanism underpinning these phenomena was found to be through MAPK/AKT pathway activation in the tumor cells. The authors also postulated that PSC-secreted factors such as interleukin-1β (IL-1β) and TGFβ were implicated in this process, the latter of which has been already correlated with gemcitabine resistance (Hesler et al., 2016). In addition, CAFs can promote irradiated cancer cell recovery and tumor relapse after RT by producing insulin-like growth factor-1/2 (IGF-1/2), C-X-C motif chemokine ligand 12 (CXCL12), and β-hydroxybutyrate (Wang et al., 2017). CXCL1 signaling is another contributor to radioresistance. Both cancer cells and CAFs express and secrete CXCL1 which then leads to ROS accumulation following RT via inhibition of the ROS-scavenging enzyme SOD1 (Alafate et al., 2020).

Stromal cell-mediated radioresistance can be also induced through direct contact-mediated signaling. In fact, PSCs promote radioprotection and stimulate the proliferation of pancreatic cancer cells through β1 integrin signaling, which is known to modulate genotoxic stress-induced cellular responses such as RT (Cordes, 2006). Notably, inhibiting β1 integrin could abolish PSC-mediated radioprotection in pancreatic cancer cells in both single-dose and fractionated RT settings (Mantoni et al., 2011). Further, other integrins have been revealed to play a role in mediating radiochemoresistance in pancreatic cancer; of which, β8 Integrin emerged as a crucial determinant and as a potential druggable target (Jin et al., 2019). Besides, the stroma may also lead to a radioresistant phenotype in pancreatic cancer by activating Akt signaling (Toulany and Rodemann, 2013).

Considering the relevance of RAS signaling in PDAC, it would be interesting to better understand how this pathway facilitates treatment resistance in this disease and how this could be exploited in the clinic. Indeed, inhibiting Ras activation could be a potential strategy for tumor-specific radiosensitization in a large majority of pancreatic cancer patients (Cengel et al., 2007; Kimple et al., 2010; Chang et al., 2014). Another likely target to alleviate resistance to therapy is ADAM9, which is overexpressed in PDAC tumors (Grutzmann et al., 2004). Notably, silencing ADAM9 could promote both radio-sensitivity and chemosensitivity in cancer cells (Josson et al., 2011). Despite all this work, again limited information is available regarding the role of specific subsets of CAFs, or their precursors for that matter, in dictating treatment responses.

## Improving Immunotherapy Response by Targeting Cancer-Associated Fibroblast Subsets

Various studies have demonstrated the complex contributions of immune cells to pancreatic cancer, and how they communicate with cancer cells as well as other members of the TME (Gorchs and Kaipe, 2021; Hanley and Thomas, 2021). An important point worth considering is that these cells may be differentially shaped depending on CAF phenotype. Most studies in this regard focus on myCAFs or iCAFs, the latter of which are hallmarked by inflammatory features. These cells are capable of secreting high levels of IL-6, which suppresses NK cell activity and leads to PDAC metastasis (Huang et al., 2019). In fact, high IL-6 levels have been previously correlated with reduced response to therapy in general and recorded to impair some ketogenic responses, thereby leading to a systemic metabolic stress response that hinders anticancer immunotherapy in PDAC (Flint et al., 2016). Following these discoveries, blocking IL-6 signaling gained momentum in the field and yielded positive preliminary findings in animal models; a combination of PD-L1 blockade and IL-6 inhibition could effectively suppress tumor progression and enhance overall survival in murine models of PDAC, which gave rise to a clinical trial (NCT04191421) adopting a similar approach. Beside iCAFs, apCAFs and csCAFs might be key stromal elements that affect immunotherapeutic approaches in PDAC as both have immunological activities as depicted above. These cells have not been studied as comprehensively as other CAF subtypes, especially in the immunotherapy department; this warrants further understanding of CAF origins and subtypes and how they differentially shape the immune system and affect therapeutic regimens.

Myofibroblasts are perhaps the most studied stromal cells in PDAC and the contributions of fibroblasts falling under the myCAF phenotype have been investigated from different angles with respect to PDAC progression and therapy response/resistance. Inhibition of TGF-β, a promoter of the myCAF phenotype, has gained some attention in the clinic and has been successfully utilized in concert with gemcitabine to improve overall survival in unresectable PDAC patients (Melisi et al., 2018). Interestingly, inhibiting TGF-β receptor can reduce IL-6 production in CAFs, leading to decreased STAT3 activation in tumors and reversed immunosuppression in mouse models (Huang et al., 2019). Further, blocking both PD-L1 and TGF-β using a double-fusion protein (M7824) inhibited tumorigenesis in mouse models (Lan et al., 2018). This shows the potential of multimodal approaches that combine PD-1/PD-L1 blockade with TGF-β inhibitors and chemotherapeutic regimens. CXC chemokines and their receptors are also relevant with respect to immunotherapy in PDAC. Indeed, CAF-secreted CXCL12 has been documented to induce an immunosuppressive environment in PDAC tumors; blocking the effect of CXCL12 on PDAC cells could enhance antitumor immunity (Garg et al., 2018). Besides, inhibition of CXCR4, using AMD3100, in combination with PD-L1 blockade induced T-cell accumulation in a KPC mouse model leading to reduced cancer proliferation (Feig et al., 2013). The COMBAT trial, a phase IIa clinical trial, was recently conducted to evaluate the efficacy and safety of the CXRC4 antagonist BL-8040 with pembrolizumab and chemotherapy in metastatic PDAC (NCT02826486). The results showed that combined CXCR4 and PD-1 blockade expanded the benefit of chemotherapy in PDAC (Bockorny et al., 2020). Moreover, the CXCL3-CXCR2 axis could stimulate the transformation of CAFs to myCAFs, which secrete type III collagen and accelerate tumor metastasis (Sun et al., 2021). In fact, inhibition of CXCR2 and CCR2 could reverse tumor progression promoted by type I collagen deletion in myCAFs as was evident in a PDAC mouse model potentially by cytotoxic T cell trafficking (Chen Y. et al., 2021). It is worth noting that both CXCR2 and CCR2 promote infiltration of suppressive myeloid cells as well. The CCR2/CCL2 axis also plays a particularly important role in attracting monocytes, which, after interactions with tumor- and stromal-derived factors, differentiate into suppressive tumor-associated macrophages at the site (Nakaoka et al., 2021). A combined blockage of CCR2 and CXCR2 in a murine PDAC model prevented CCR2+ macrophages (Burfeind et al., 2020); this approach may also reduce the number of stellate cells and ultimately CAFs in the TME as CCR2 signaling is involved in monocyte recruitment to PDAC tumors (Ino et al., 2014).

As outlined earlier, SHH signaling is important to consider in PDAC also from an immunological perspective. Initially, it was shown that depletion of CAFs could induce immunosuppression in PDAC tumors with indications that the Hedgehog pathway may play a role in this phenomenon (Rhim et al., 2014; Özdemir et al., 2014). This is in accordance with previous literature that describes the role of SHH in shaping the immune environment (Bai et al., 2016). A recent paper provided more insight as to why Shh inhibition is complex in PDAC treatment (Steele et al., 2021). It was evident in this study that inhibiting Shh signaling using the Smoothened antagonist LDE225 decreases the myCAF/iCAF ratio in the tumor stroma, resulting in an immune suppressive microenvironment. The observed effects were linked to a decrease in the number of cytotoxic T cells and an increase in that of regulatory T cells, as occurs in an immune-suppressive environment, and suggests that targeting SHH would be counterintuitive in PDAC patients.

Another study also explored the premise of altering the fibroblast composition as a therapeutic strategy for PDAC. It was identified that tumor-secreted IL1, predominantly through autocrine LIF, activates the JAK/STAT pathway in CAFs (Biffi et al., 2019). Subsequently, JAK/STAT signaling maintains an inflammatory CAF phenotype through a positive feedback loop involving STAT3-mediated upregulation of IL1R1. Interestingly, however, treating tumor-bearing KPC mice with the JAK inhibitor AZD1480 led to a significant decrease in cancer cell proliferation and tumor growth as well as a significant increase in collagen deposition; It was also apparent that AZD1480-treated tumors had increased levels of αSMA, suggesting that JAK inhibition may promote a shift from an iCAF phenotype toward a myCAF-like state (Biffi et al., 2019). This approach was deemed likely to improve therapeutic outcomes in PDAC patients as it simultaneously targets potential tumor-promoting components, such as iCAFs, along with components that impede drug delivery, such as myCAF-derived desmoplasia. Nonetheless, further research should be performed to establish safe and effective fibroblast altering strategies for the clinic.

Together, the abovementioned studies introduce some of the pathways by which CAFs are involved in response to immunotherapy and underline the importance of targeting select CAF subsets or certain pathways that underpin resistance to immunotherapy rather than using general stromal disruptive agents or targeting the entire CAF population.

## The Involvement of Cancer-Associated Fibroblasts in Pancreatic Cancer Metastasis

Metastatic dissemination is a process that is heavily reliant on stromal cues and tumor-stroma communication (Joyce and Pollard, 2009). CAFs can promote the invasiveness of cancer cells as well as angiogenesis by secreting a plethora of growth factors and extracellular matrix molecules. CAF-mediated ECM deregulation may lead to biomechanical and biochemical changes to the TME, thereby facilitating tumor cell invasion and metastasis (Cao et al., 2016; Glentis et al., 2017). Moreover, PDAC cells can educate CAFs and drive them into establishing a prometastatic microenvironment; these fibroblasts contribute to the formation of a niche that supports the survival and expansion of extravasated cancer cells (Brodt, 2016; Houg and Bijlsma, 2018). In one study, weakly metastatic cancer cells stimulated co-cultured MSCs, a source of CAFs, into secreting the chemokine CCL5, thereby promoting the invasion of the cancer cells and metastasis (Makinoshima and Dezawa, 2009). Nonetheless, it is not yet clear whether all CAFs contribute to the metastatic dissemination of PDAC cells. One group identified a population of matrix-remodeling CAFs expressing the Endo180 (*MRC2*) receptor that supported tumor growth and metastasis (Jungwirth et al., 2021).

The paired-related homeobox 1 (Prrx1) transcriptional factor is a key regulator of epithelial-to-mesenchymal transition (EMT) and metastatic colonization in PDAC. Prrx1 is also highly expressed in PDAC stroma and was reported to mediate CAF activation, leading to increased ECM deposition, improved tumor differentiation, fewer circulating tumor cells, and reduced metastasis (Feldmann et al., 2021). CAFs expressing Prrx1 could promote EMT and chemotherapeutic resistance in tumor cells through paracrine HGF signaling. It is also noteworthy that high Prrx1 expression was correlated with the squamous subtype, whereas low stromal Prrx1 expression was predominant in classical tumors, which may indicate differences in stromal content between the two subtypes. Other studies have shown that overexpression of ETV1, another transcription factor, drastically increases the incidence and volume of micro- and macro-metastases in mouse models through stromal expansion (Heeg et al., 2016).

Besides, characterizing stroma within metastatic lesions in an autochthonous model of PDAC indicated that myofibroblasts appear when metastases are as small as 6–7 cells and that cell populations within these lesions become more epithelial during growth (Aiello et al., 2016). Interestingly, fibroblasts at metastatic sites differ from CAFs within primary tumors and are often termed metastasis-associated fibroblasts (MAFs); MAFs make significant contributions to the establishment of pre-metastatic niches and, subsequently, metastatic lesions and encourage therapeutic resistance in metastatic tumors. These cells are capable of remodeling the extracellular matrix of metastatic tumors, modulating immune cells in the tumor microenvironment, promoting angiogenesis and enhancing malignant tumor phenotypes (Wang et al., 2021). MAFs in liver metastases of pancreatic cancer seem to promote angiogenesis and resistance to antiangiogenic drugs through secretion of CCL2 and CXCL8; preclinical studies suggest that targeting MAFs can alleviate the progression of metastatic cancer and mitigate therapeutic resistance (Pausch et al., 2020). Indeed, others have also demonstrated the crucial role the immune system plays in the process of PDAC metastasis. A study showed that the regulation of stroma within PDAC liver metastases is unique and dependent on immune interactions, which may precede cancer cell metastasis. They further demonstrated that metastasis-associated macrophages (MAMs) derived from bone marrow cells rather than native Kupffer cells; in contrast, metastasis-associated fibroblasts were found to be of local origin, presumably hepatic stellate cells, which raises some questions regarding the cellular origins of CAFs in distant metastases compared to primary tumors (Nielsen et al., 2016; Quaranta et al., 2018). Nielsen et al. (2016) also showed that chemical ablation of MAMs in mice after metastatic seeding had occurred was sufficient to decrease the accumulation of activated myofibroblasts as well as reduced the size of the area covered by metastatic cells; although, it did not significantly reduce the metastatic frequency. They went further to identify that macrophage-conditioned media could activate quiescent fibroblasts through granulin, which was, remarkably, only expressed in bone marrow-derived macrophages found in liver metastases and not in those found at the primary tumor site, raising some questions that warrant further studies. The CAF-tumor-associated macrophage (TAM) axis is a major player in PDAC metastasis. In fact, CAFs produce high levels of IL-33 that induces an M1-to-M2 transition in TAMs, which then exhibit elevated levels of MMP9; in mouse and human fibroblast-rich pancreatic tumors, genetic deletion of IL-33 or MMP9 markedly blocked metastasis (Andersson et al., 2018; Yang et al., 2021).

One paper eloquently introduces the processes involved in the metastatic dissemination of pancreatic cancer cells (Shan et al., 2017). It was described that CAFs activated through paracrine Hedgehog signaling in turn induce the Snail transcription factor in PDAC cells, thereby leading to EMT in the cancer cells (as indicated by vimentin upregulation and E-cadherin downregulation) and enhancing their invasive capacity. It was also hypothesized that after circulating tumor cells home into a new environment, the paracrine action of normal non-activated fibroblasts downregulates this axis in the cancer cells, leading to the formation of new metastatic foci, which in turn activate CAFs and initiate a new cycle.

Together, the abovementioned studies highlight the importance of CAFs in pancreatic cancer metastasis and illustrate the need for more comprehensive studies on the cellular origins of CAFs or MAFs in PDAC metastases and how they phenotypically differ from their counterparts in primary sites.

## The Effects of Cancer Therapies on Cancer-Associated Fibroblasts

Another aspect that is important to consider is treatment-induced changes to the TME. During the process of being activated, and in response to therapeutic regimens, CAFs undergo changes that grant them resistant characteristics; this mainly occurs through a defective p53/p21 response pathway and high expression of the cancer marker Survivin (Hawsawi et al., 2008; Arandkar et al., 2018; Wang et al., 2019). Since the stroma presents the larger fraction in PDAC tumors, and taking into account stroma-driven resistance, it is not unlikely that this adds yet another obstacle for the delivery of therapeutic regimens.

Cancer-associated fibroblasts and mesenchymal stem cells have been consistently shown to be enriched in chemotherapy-treated human tumors, including PDAC, wherein they promote cancer growth and treatment resistance by secreting various paracrine factors (Chan et al., 2019). Not only that, but exposing these cells to cytotoxic agents seems to also alter them toward a senescence-like secretory phenotype that encourages stemness features and aggressiveness in the surrounding cancer cells (Lotti et al., 2013). Similarly, quiescent PSCs have also been recorded to undergo a phenotypic and functional transition toward an activated myofibroblast state in response to noxious agents such as alcohol (Charrier et al., 2014). As such, it would not be too farfetched to assume that this phenomenon also takes place when cytotoxic agents and radiation regimens are introduced. Considering the fact that a highly dense stromal compartment supports cancer cell resistance by providing a mechanical barrier that diminishes the potency of anticancer drugs (Miao et al., 2015), the contributions of stellate cell activation to PDAC progression should be fully understood.

Molecular analysis-based studies on neoadjuvant chemotherapy–treated human PDAC tumors and orthotopic tumor xenografts revealed that traditional, or maximum-tolerated dose, chemotherapy regimens induce persistent STAT-1 and NF-κB activity in CAFs, subsequently resulting in high expression and secretion of ELR motif–positive (ELR^+^) chemokines. On the contrary, administering the same overall dose of a certain drug over a longer time frame, as a low-dose metronomic chemotherapy regimen, largely reduced therapy-induced stromal ELR^+^ chemokine paracrine signaling and, thus, enhanced treatment response and improved mouse survival rates (Chan et al., 2016).

Chemotherapy-induced oxidative stress plays a controversial role in cancer and has raised some concerns regarding the effectiveness of combinatorial approaches (Shacter et al., 2000; Liu and Wang, 2015; Li et al., 2020). A recent study suggested that oxidative stress could be another contributor to PDAC desmoplasia in the sense that it can induce p38-mediated monocyte-to-myofibroblast transdifferentiation (MMT), thereby leading to stromal activation, modulating immunosuppression, as well as promoting tumor progression (Huang et al., 2020). This contributes to the uncertainty regarding the use of oxidative stress-inducing agents in cancer treatment and urges more comprehensive studies.

An important consequence that may take shape because of RT is chronic inflammation that drives fibrosis and leads to an increase in stromal members in the TME as well as more ECM components (Straub et al., 2015). CAFs tolerate relatively high doses (30 Gy) of radiation without apoptosis; however, doses higher than 10–12 Gy often result in a senescent CAF phenotype (Ragunathan et al., 2020). Premature senescence seems to be also induced in normal human fibroblasts as a result of chronic low dose rate (LDR) exposure (5 or 15 mGy/h) of gamma rays (Fujimori et al., 2005). In addition to inducing premature cellular senescence, exposing CAFs to RT results in the potent induction of multiple DNA damage response (DDR) foci as well as the inhibition of the proliferative, migrative, and invasive capacity of these fibroblasts (Goel et al., 2013; Li et al., 2018; Im et al., 2020). Senescent CAFs have been described to present a senescence-associated secretory phenotype that is characterized by the upregulation and secretion of various substances (e.g., CXCL12, TGF-β1, IGF-1, IGFBP2, and NO) (Li et al., 2016; Ansems and Span, 2020), some of which are pro-tumorigenic factors, such as IL-6, IL-8, and osteopontin, and are linked to stroma-mediated therapeutic resistance (Krisnawan et al., 2020). Various cytokines such as TGF-β1, TNF-α, IL-1, IL-4, and IL-13; chemokines such as MCP-1 and MIP-1β; as well as angiogenic and growth factors are involved in RT-induced fibrosis (Ansems and Span, 2020).

## Next Steps for PDAC Modeling

In the previous part of this review, we focused on the concept of CAF heterogeneity in PDAC and how CAF subsets may have functionally different roles in the tumors. We also discussed how CAFs behave in response to tumor-secreted factors as well as therapeutic regimens and raised some concerns regarding the potential heterogeneous responses of different CAF subsets. Indeed, we would like to re-emphasize the importance of considering CAF heterogeneity, which may be facilitated by CAF cellular origins, when designing targeted therapeutic regimens for PDAC patients to avoid unwanted contraindications (Öhlund et al., 2017; Elyada et al., 2019). Of course, the dose and frequency of regimens likely affect therapy response and CAF diversity and senescence, and should be further investigated. Despite all this information, we are still limited in our understanding of tumor-stroma interactions and CAF-induced changes on tumor progression as well as chemo- and radio-resistance. We believe that considerations and improvements should be made to establish advanced *ex vitro* tools for effectively modeling PDAC and revamping therapeutic strategies. Therefore, in the next section of this paper, we will suggest pre-clinical models that might be useful for modeling PDAC as well as recapitulating CAF heterogeneity and the processes of communication that take place within these tumors.

## The Contribution of Pre-Clinical Models to Modeling Tumor-Stroma Interactions

For a long time, animal models and conventional two-dimensional (2D) cell culture systems have been used to study and understand human pathology. However, despite the fact that these models led to various scientific advances, their utility for modeling human physiology and pathology is limited for several reasons (Velasco et al., 2020). Hence, it is quite evident that there is a need for advanced *in vitro* or *ex vivo* tools to model desmoplastic diseases, especially those with complex stromal dynamics like PDAC.

To fill this gap, effort has been invested to establish more advanced technologies in order to generate 3D human tissue-like models; this led rise to the current systems we know as organoids and spheroids (Costa et al., 2016; Kim et al., 2020). Subsequently, many modifications have been made to improve and/or adapt these 3D models for different cancer types. In the context of pancreatic cancer, organoid-based models gained the most attention and have since become a standard for modeling the disease (Baker et al., 2016). Nonetheless, despite the success of organoids in recapitulating pancreatic cancer, the lack of stromal components in these models is still a major obstacle that limits their clinical worth. Indeed, most organoids lack the characteristic fibrotic stroma that is a major obstacle for drug delivery as well as a crucial abettor for cancer cells as was evident throughout this review. Further, inclusion of immune cells in *ex-vivo* culture systems is a critical step to establish a platform for the study of immunotherapy in pancreatic cancer (Tiriac et al., 2019). Fundamentally, a robust patient-matched co-culture system is the optimal approach for researchers and clinicians to model PDAC and assess various therapeutic strategies before proceeding to the clinical setting.

To that end, many groups sought out to establish co-cultures of organoids and stromal cells including resident fibroblasts, cancer associated fibroblasts, pancreatic stellate cells, and immune cells (Hwang et al., 2019). The first move toward this goal arose in the form of combining pancreatic organoids/spheroids with CAFs or PSCs which led to valuable advances in studying invasion, matrix remodeling, and drug response (Khawar et al., 2018; Che et al., 2020). Nonetheless, there was still a shortage of models that took into account the immune microenvironment and which allowed co-culturing a number of different cell types. Recently, more progress has been possible due to technological advances in biomimetic scaffolds, 3D bioprinting techniques, as well as microtechnology-based systems (Sun et al., 2019). Indeed, some microfabricated organoid models, which are generated through the use of techniques and methods implemented in the development of microelectromechanical systems, such as micropatterning and microfluidics have the potential to transform organoid production (Velasco et al., 2020). These new methods are of great utility to the cancer field as they allow us to culture several cell types together and take one step closer toward truly recapitulating the *in vivo* setting (Velasco et al., 2020). Recently, many microfluidic 3D platforms were described for culturing PDAC. One study provided evidence that PDAC cells can be cultured on the HepaChip, a novel microfluidic chamber, which maintains cell vitality, morphological appearance, and growth characteristics (Beer et al., 2017). The group argues that their microfluidic system allows for continuous perfusion, which is where previous models have failed. Moreover, more advanced strategies tried to combine organ-on-a-chip and organotypic technologies to better emulate the vascular aspect of PDAC tumors. One group presented an organotypic PDAC-on-a-chip culture, featuring a 3D matrix containing juxtaposed PDAC and perfusable endothelial lumens, which emulate vascular invasion and tumor–blood vessel interactions (Nguyen et al., 2019). An arguably even more interesting concept is maintaining primary-derived tissue in short culture for downstream analyses. A recent study described a promising pre-clinical model of PDAC that maintains the viability, 3D multicellular architecture and microenvironmental cues of unmanipulated patient derived tumors (Kokkinos et al., 2021). The group presented a simple and cost-effective model that bridges the gaps and provides unprecedented opportunity to closely study the biology of pancreatic cancer; of course, such models are quite valuable for identifying novel therapeutic targets involved in tumor-stroma crosstalk as well as testing new combinatorial treatment regimens. In parallel, other groups have focused on 3D bioprinting approaches to design miniaturized tumor models that are capable of self-organization (Langer et al., 2019); one spheroid-based array showed great promise for studying the formation of precursor PDAC lesions and cancer progression (Hakobyan et al., 2020).

Taken together, the abovementioned tools present good possibilities for modeling PDAC. Considering the dynamic and heterogeneous nature of PDAC tumors, it is essential to use advanced models that allow for co-culturing cancer cells with different members of the stroma in order to recreate the *in vivo* environment and identify precious biomarkers for targeted therapies. It has become common knowledge in the field that CAFs have different cellular sources and that they can have different functions within a PDAC tumor depending on interactions with cancer cells and other factors. Indeed, there are indications that CAFs behave, or are influenced, differently depending on geographical factors and culture conditions such is the case in the work of Öhlund et al. (2017) and a more recent study on AD-MSC differentiation into iCAFs vs. myCAFs (Miyazaki et al., 2021). Therefore, to be able to effectively and accurately model pancreatic cancer, we are in need of models that can (1) maintain the morphological and growth characteristics of parent tumors as well as their mutational profiles; (2) the models should also take non-cancer compartments into account by including most of the stromal cell types that are present in the *in vivo* setting; and (3) be able to recapitulate the phenotypes and functionality of different CAF populations and subsets in PDAC tumors. Of course, this is essential to allow proper drug response predictions as the stroma is one of the main obstacles for drug delivery as well as an important contributor to resistance in pancreatic cancer.

## Limitations and Concluding Notes

It has become apparent throughout this paper how important CAFs are in PDAC tumors and how complex and seemingly opposing their contributions can be. It is for this reason that we would like to stress the urgency of more advanced studies that focus on the cellular origins of CAFs, how these precursors contribute to the CAF population, and how this drives stromal heterogeneity in PDAC. We are also in need of markers that can be used to accurately identify different CAF subsets and their potential function, possibly as potential biomarkers to select for (novel) targeted therapeutic approaches. It might, thus, be relevant to investigate and better understand the gradual variations in the stromal profile during PDAC progression. In order to make headway on this matter, we believe that more advanced *ex vivo* models need to be implemented in combination with lineage tracing and scRNA-seq technology. This combination might not be currently practical or feasible in light of technological limitations; however, this does not reduce from value of establishing and implementation such platforms in the near future. In conclusion, we believe that accurately modeling PDAC and unraveling the paradigm that is CAF heterogeneity are crucial for reaching clinically relevant findings and making strides toward personalized or targeted treatment approaches.

## Author Contributions

PM drafted the manuscript and figures. HL and MB provided supervision and revised the manuscript. All authors have read and accepted the final version of the manuscript.

## Conflict of Interest

PM has nothing to disclose. MB has received research funding from Celgene and reports an advisory role for Servier outside the scope of the submitted work. HL reports grants from Bristol-Myers Squibb, grants from Bayer Schering Pharma, grants from Celgene, grants from Janssen-Cilag, grants from Lilly, grants from Nordic Group, grants from Philips Healthcare, grants from Roche, grants from Merck Sharp and Dohme, personal fees from Lilly, personal fees from AstraZeneca, personal fees from Lilly, personal fees from Nordic, personal fees from Bristol-Myers Squibb, outside the scope of the submitted work.

## Publisher’s Note

All claims expressed in this article are solely those of the authors and do not necessarily represent those of their affiliated organizations, or those of the publisher, the editors and the reviewers. Any product that may be evaluated in this article, or claim that may be made by its manufacturer, is not guaranteed or endorsed by the publisher.

## Abbreviations

Heterogeneity: The quality or state of elements being diverse or dissimilar
Phenotype: The total of all observable properties including morphological and functional aspects
Population: The total number of members in a particular area that are present at the same time
Subset: A group of unique elements/members that are contained within a population
Stellate cells: Typically quiescent fibroblast-like cells found in the liver or pancreas that are involved in tissue fibrosis
Fibroblasts, A connective-tissue cell of mesenchymal origin that secretes proteins: especially molecular collagen from which the extracellular fibrillar matrix of connective tissue forms
Cancer-associated fibroblasts: Constitutively activated fibroblasts that reside within a tumor
Mesenchymal stem cells, Multipotent cells isolated from various organs that are able to proliferate and self-renew: as well as to give rise to progeny of at least the osteogenic, chondrogenic and adipogenic lineages.
